# FSI simulation of CSF hydrodynamic changes in a large population of non-communicating hydrocephalus patients during treatment process with regard to their clinical symptoms

**DOI:** 10.1371/journal.pone.0196216

**Published:** 2018-04-30

**Authors:** Seifollah Gholampour

**Affiliations:** Department of Biomedical Engineering, Islamic Azad University–Tehran North Branch, Tehran, Iran; Medical Photonics Research Center, Hamamatsu University School of Medicine, JAPAN

## Abstract

3D fluid-structure interaction modelling was utilized for simulation of 13 normal subjects, 11 non-communicating hydrocephalus (NCH) patients at pre-treatment phase, and 3 patients at five post-treatment phases. Evaluation of ventricles volume and maximum CSF pressure (before shunting) following results validation indicated that these parameters were the most proper hydrodynamic indices and the NCH type doesn’t have any significant effect on changes in two indices. The results confirmed an appropriate correlation between these indices although the correlation decreased slightly after the occurrence of disease. NCH raises the intensity of vortex and pulsatility (2.4 times) of CSF flow while the flow remains laminar. On day 18 after shunting, the CSF pressure decreased 81.0% and all clinical symptoms of patients vanished except for headache. Continuing this investigation during the treatment process showed that maximum CSF pressure is the most sensitive parameter to patients’ clinical symptoms. Maximum CSF pressure has decreased proportional to the level of decrease in clinical symptoms and has returned close to the pressure range in normal subjects faster than other parameters and simultaneous with disappearance of patients’ clinical symptoms (from day 81 after shunting). However, phase lag between flow rate and pressure gradient functions and the degree of CSF pulsatility haven’t returned to normal subjects’ conditions even 981 days after shunting and NCH has also caused a permanent volume change (of 20.1%) in ventricles. Therefore, patients have experienced a new healthy state in new hydrodynamic conditions after shunting and healing. Increase in patients’ intracranial compliance was predicted with a more accurate non-invasive method than previous experimental methods up to more than 981 days after shunting. The changes in hydrodynamic parameters along with clinical reports of patients can help to gain more insight into the pathophysiology of NCH patients.

## 1. Introduction

**Fig 1:**
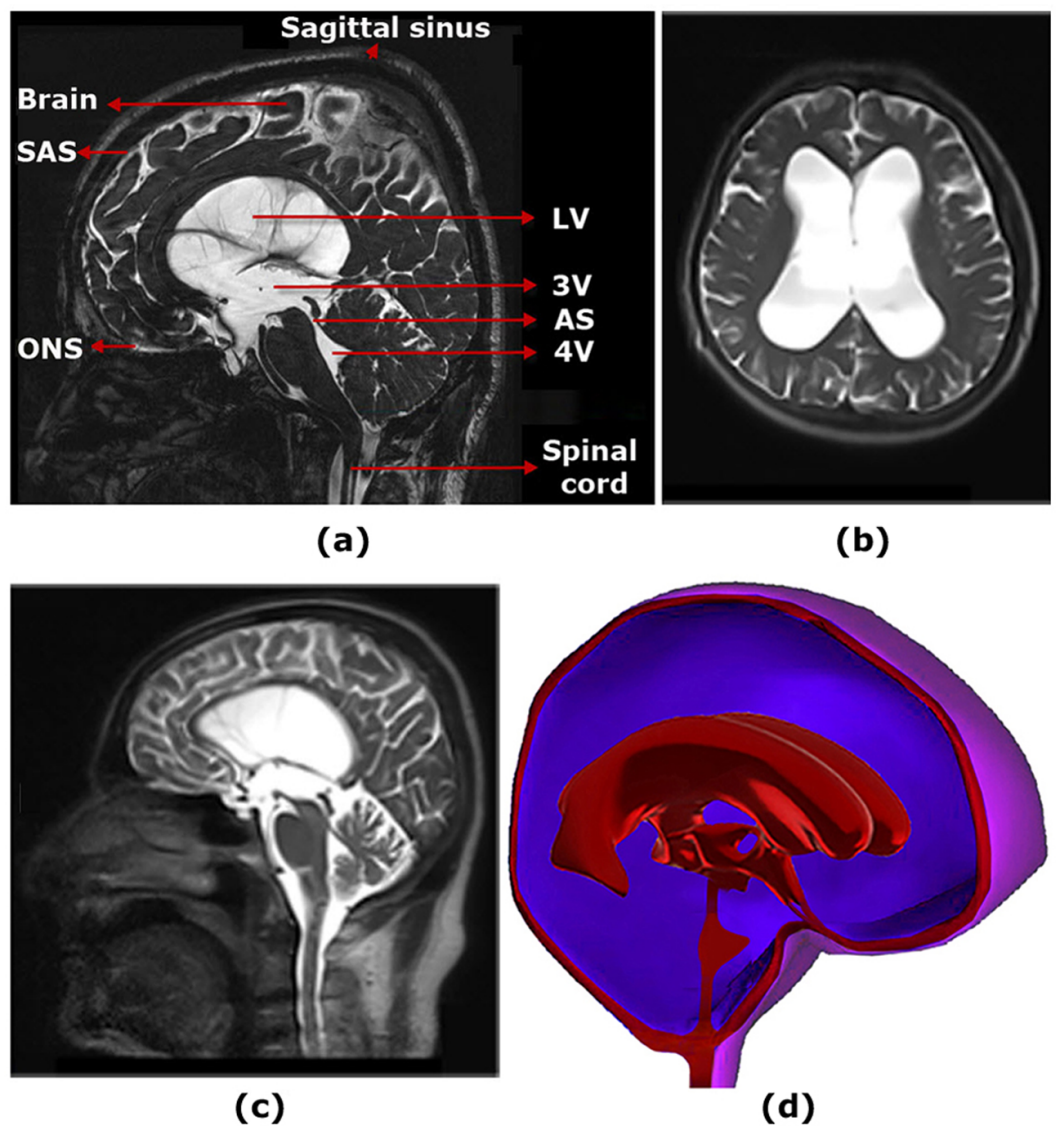
(a) Illustrates the detailed information about locations of CSF production and absorptions and brain tissue in a hydrocephalus patient. (b) and (c) show respectively the axial and sagittal planes of MRI images of patient No.1 before shunting. (d) Shows the 3D model of the ventricular system, brain tissue and subarachnoid space of patient No.1 before shunting. LV: lateral ventricle; 3V: third ventricle; 4V: fourth ventricle; SAS: subarachnoid space; AS: aqueduct of Sylvius; ONS: optic nerve sheath.

The production of cerebrospinal fluid (CSF) is the primary function of the choroid plexuses [1]. Active CSF space expansion is defined as hydrocephalus caused by disturbance in CSF pathway from production to absorption locations (Fig 1a). Choroid plexus also acts as the regulator of CSF pressure during hydrocephalus development [2]. Tumour or obstruction/blockage in the ventricular CSF pathway is described as the cause of non-communicating hydrocephalus (NCH) in most cases [3]. It is worth mentioning that pathophysiology of NCH is less known [3]. The reason can be related to the lack of knowledge about changes in CSF hydrodynamic parameters during disease occurrence and its treatment process. Several studies have been conducted to alleviate these knowledge shortcomings. These studies can be divided into two general groups.

The first group includes experimental studies on hydrocephalus patients during their treatment procedures which evaluate and compare CSF hydrodynamics during treatment process [4–9]. Most of the experimental methods used in these studies for measuring the hydrodynamic parameters were invasive methods which are always accompanied by risks and restrictions. For instance, cine phase contrast magnetic resonance imaging (cine-PC MRI) has restriction in measurement of CSF velocity and flow rate in all CSF spaces. It also lacks the ability of CSF pressure measurement [10]. Measuring CSF pressure at all locations, such as behind optic nerve sheath (BONS), is not also easily possible through experimental methods, while some clinical symptoms of hydrocephalus patients are related to impaired vision and papilledema which are caused by an increased CSF pressure in BONS [11]. The use of some methods such as lumbar puncture to measure the CSF pressure in NCHs is also accompanied by many limitations [12]. Hence, the use of computer modeling, which calculates all hydrodynamic details of CSF at all locations non-invasively, doesn’t involve any of the problems related to experimental methods although it is regarded as a time consuming method.

The second group includes those studies which evaluate CSF flow using computer modeling. In this group, there are also studies using 2D computer simulations or studies with incomprehensive solution methods like finite element method or computational fluid dynamic for modeling hydrocephalus [13–17]. They have limitations in evaluating hydrocephalus conditions precisely since 2D analysis cannot express all geometric complexities of head and cannot yield a precise solution. Further, finite element method disregards the complexities of CSF flow and computational fluid dynamic method doesn’t consider the deformability of the walls in the ventricular system which plays a significant role in changing the volume of ventricles and brain that leads finally to hydrocephalus. Therefore, the 3D fluid-structure interaction (FSI) modeling is the most proper method to analyze the CSF flow in hydrocephalus patients since it has none of those limitations and problems.

Some studies compared the CSF flow among normal subjects and hydrocephalus patients (before treatment) using FSI simulations [10,11,18]. Vardakis et al. evaluated the efficiency of endoscopic third (ETV) and endoscopic fourth ventriculostomy in treatment of hydrocephalus patients using computer modeling [19]. Tangen et al. simulated the cranial and spinal model of the CSF with fluid motion using FSI method for evaluating the spinal microanatomy effect on CSF [20]. They also modeled the changes in CSF and intrathecal drug flow dynamics in treatment of central nervous system diseases and evaluated some parameters affecting the fluid circulation using CFD and FSI methods [21–23]. In the recent study, Gholampour et al. considered merely the manner of changes in the effective parameters and clinical symptoms of the disease before the beginning of the treatment process of hydrocephalus patients [11]. However, none of the previous studies evaluated the details of the hydrodynamic parameters changes of the CSF flow during the treatment process of the NCH patients treated by shunting using computer simulation. One of the less known concerns, which hasn’t been investigated in previous studies, is the question whether the biofluid parameters of CSF returns after patients’ healing to the normal conditions of healthy individuals or which parameters return to which level. This study is an attempt to find answer to these questions using 3D FSI modeling up to 2.5 years after treatment of NCH patients.

## 2. Materials and methods

### 2.1. Case population and treatment method

Eleven NCH patients who experienced no disruption in their shunt during treatment process were recruited. As no record of patients’ conditions before disease was available, 13 normal subjects of approximately the same sex, age, height and weight were selected in order to compare the deviation of patient conditions from normal status. Relevant information on NCH patients and normal subjects are listed in Table 1. Despite the fact that ETV and ventriculoperitoneal shunt (VPS) are among the common treatments of NCH [24], the optimal treatment for NCH patients is still unknown [4]. Dewan et al. indicated that ETV failure has taken place sooner than VPS failure [4]. In addition, Eide et al. found it somewhat impossible to prove ETV as a better treatment than VPS for NCH [3]. Regarding the patients’ conditions based on diagnosis of the medical team, VPS with differential pressure valve (Cordis-Hakim standard system) was used in the present study for treating the NCH patients. The shunt was placed in one-way format in patients’ right lateral ventricle.

**Table 1:** Relevant information on NCH patients and normal subjects: Age (year), height (m), weight (kg), heart rate (beat/min), gender, head substructure’s volume (ml), maximum CSF velocity (cm/s), and Reynolds number of 11 pre-shunting non-communicating hydrocephalus patients and mean values of these values of 13 normal subjects. NCH: Non-communicating hydrocephalus; LVs: lateral ventricles; 3V: third ventricle; 4V: fourth ventricle; SAS: subarachnoid space; AS: aqueduct of Sylvius; SD: standard deviation; NS: normal subject.

Case number	NCH 1	NCH 2	NCH 3	NCH 4	NCH 5	NCH 6	NCH 7	NCH 8	NCH 9	NCH 10	NCH 11	13 NS Mean ±SD
Height	1.71	1.53	1.75	1.62	1.63	1.57	1.68	1.57	1.68	1.62	1.51	1.64±0. 2
Weight	72	58.7	79.2	60.2	71.3	64.4	69	67.8	75.2	68.2	60.1	70.6±10.7
Age	59	57	69	67	76	54	59	71	64	76	74	64.9.4±7
Heart rate	82	82	71	73	68	74	70	81	77	90	69	74.8±5.7
Gender	Male	Female	Female	Male	Male	Female	Male	Female	Male	Male	Female	---
Cause of NCH*	AS	AS	AS	AS	AS	MT	MT	MT	AW	AW	AW	---
Clinical symptoms **	A-E, G	C-E	A-E, G	B-F	D-F	H-J, L	E, G- J, L	F, G, H, L	B, F-I	B, G-I, L	A, F-I, L	---
Volume—LVs	269.4	277.8	285.6	234.1	261.8	248	258.1	270	270	257.2	281.3	11.80±0.52
Volume -3V	11.8	12.5	10.7	10.9	11.7	12.3	10.5	12.2	12	12.7	12.5	4.7±0.2
Volume -4V	5.1	4.8	4.7	4.3	4.5	5.2	4.4	4.8	4.6	5.1	5	3.9±0.2
Volume -SAS	120.1	112.8	113.1	98.3	105.3	98.6	101.5	108.1	108	116.7	109.8	105.1±4.3
Volume -Brain	1102.8	1084.7	1098.7	988.6	1070.8	1009.2	990	1081.6	1173.4	993.2	1190.7	1207.1±50
MaximumCSF velocity -AS	9.82	8.13	8.52	5.01	5.48	4.01	4.41	6.72	5.46	5.11	5.51	3.92±1.3
Reynoldsnumber -AS	468.3	443.7	378.4	354.7	358.7	352.9	359.3	353.9	363.9	363.8	374.3	310.8±33.4

* Cause of NCH: AS: Aqueductal stenosis; MT: Mesencephalic tumour; AW: Aqueductal web.

** Clinical symptoms: A: sleepiness; B: nausea and vomiting; C: headache; D: seizures; E: papilledema; F: balance and gait disturbances; G: urinary incontinence; H: hemiparesis; I: upward gaze paresis; J: gait disturbance; K: mental impairment; L: nausea and vomiting.

It is noteworthy that the number of recruited cases is regarded as one of the advantages of this study. In previous computer simulations, fewer numbers of cases comparing to the present study were recruited although they did not evaluate the treatment and healing process of patients with shunt surgery using FSI. Hence, the results of this study are more reliable.

All procedures performed in studies involving human participants were in accordance with the ethical standards of North Tehran Branch, Islamic Azad University, Tehran, Iran, (Ethics committee of biomedical research center) and with the 1964 Helsinki declaration and its later amendments or comparable ethical standards. Furthermore, this article does not contain any studies with animals performed by any of the authors.

### 2.2. Cine PC-MRI finding and model development

The cine-PC MRI is considered as a tool for *in vivo* measurement of CSF flow. The velocity encoding value, repetition time, flip angle, echo time, field of view, slice thickness and matrix size were 100cm/s, 18msec, 23°, 8.3msec, 23cm, 3mm and 256x198, respectively. The details of this imaging protocol were provided in the previous study [25]. The cine-PC MRI was performed on 11 NCH patients and 13 normal subjects (Fig 1b and 1c) by a 3-Tesla MRI unit (Magnetom Trio, Siemens, Erlangen, Germany). The first output of cine-PC MRI was CSF velocity in aqueduct of Sylvius (AS), which was used for comparing and validating the calculated CSF velocity from computer modeling (see section 3.1). The second output of cine-PC MRI was DICOM file of cases’ heads, which was transferred to the image recognition software (Mimics v13.1) to provide point clouds of the head. The 3D models of the ventricular system, brain and subarachnoid space (SAS) of 11 NCH patients and 13 normal subjects were created using their point clouds (Fig 1d). These models were then used for meshing and FSI analysis using ADINA 8.3 (Adina R&D Inc., Watertown MA, USA). Finally, the patients were treated by shunt surgery and all the above-mentioned stages were repeated on patients No.1-3 on days 5, 18, 81, 903 and 981 after shunting (the follow-up of other patients was not possible) in order to understand the changes of CSF hydrodynamics during healing process. Producing the point clouds and 3D models of the ventricular system and brain tissue of an average subject required 13 hours of manual user-input. Meshing and FSI simulation of an average subject required about 152 CPU-hours, performed by a 16-core server processor. The total time from the phase of input data collection, the required man working hours and CPU processing hours up to achieving the complete mesh convergence for results of 11 NCH patients (at one pre-treatment phase) and 3 NCH patients (at five post-treatment phases) and 13 normal subjects was about 4 years.

### 2.3. Computational analysis, material properties and boundary conditions

Fully coupled FSI simulations were performed and the arbitrary Lagrangian-Eulerian (ALE) equations were used for analyzing fluid and solid models. For iteration scheme, we used the full Newton–Raphson method. CSF circulating in the ventricles and SAS was considered as the fluid model while the brain tissue was considered as the solid model. Due to insignificance of skull deformation in hydrocephalus patients [26], the outer layer of SAS (inner layer of the skull) was constrained in three directions. The brain outer and inner surfaces in contact with CSF were assumed as FSI boundary conditions, in which the displacement compatibility and traction equilibrium were considered according to the previous literatures [10,27]. It should be noted that one of the most complex parts of this modeling was the separation of the SAS and brain outer surfaces. The layers were obtained manually using the image reconstruction technique in the Mimics software.

Further, the CSF was assumed as an isothermal, incompressible Newtonian fluid. The dynamic viscosity and density of CSF equaled to 0.001 kg/ms and 1000 Kg/m^3^, respectively [10,11,13]. The following equations governed the fluid flow [10,13]:

$$\nabla .u_{F}=0$$ (1)

$$\rho_{F}(\nabla .u_{F})=S^{f}$$ (2)

$$\rho_{F}\frac{\partial u_{F}}{\partial t}+\rho_{F}((u_{F}-W).\nabla )u_{F}=-\nabla p+\mu \nabla^{2}u_{F}+f_{F}^{B}$$ (3)

where μ, u_F_, W, P, S^f^, ρ_F_ and $f_{F}^{B}$ represent the CSF dynamic viscosity, CSF velocity vector, moving mesh velocity vector, CSF pressure, constant CSF production, CSF density and force per unit volume, respectively. "u_F_-W" in ALE represents the relative velocity of CSF regarding the moving coordinate velocity. Eqs (1) and (2) represent the CSF production in SAS and ventricles, respectively and Eq (3) is the momentum equation [13]. The numerical simulation applied for the brain tissue (solid model) was formulated based on the Lagrangian model [28]:

$$\nabla .\sigma_{S}+f_{F}^{B}=\rho_{S}{\ddot{u}}_{S}$$ (4)

where ρ_s_, *σ*_s_ and *ü*_*S*_ represent the brain density, Cauchy’s stress tensor of brain and the local acceleration of the solid part, respectively.

Previous studies have used various constitutive models to analyze the brain tissue in hydrocephalus patients. According to the study by Hrapko et al. the brain tissue should be analyzed with a time-dependent stress-strain model [29]. Cheng et al. considered the brain tissue as a poro-viscoelastic material for analyzing hydrocephalus patients [26] and their results were consistent with those of previous experimental studies. Accordingly, the poro-viscoelasticity assumption was applied for brain tissue in the present study.

The general equations were derived from the equation of the solid model, law of Darcy of fluid flow through a porous medium and the conditions of equilibrium of a stress field according to the study by Cheng et al. The additional parameters in a poro-viscoelastic model include the modulus of relaxation [26].The constitutive relation of the viscoelastic solid phase was expressed by Prony series [26]. Time dependent shear relaxation modulus equation is expressed as below:

$$Gr(t)=G_{0}(1-\sum_{k=1}^{N}g_{k}^{-p}{(1-e}^{-(\frac{t}{\tau_{k}})})$$ (5)

G_o_ represents instantaneous shear modulus.*τ*_k_ and $g_{k}^{p}$ are the input parameters of the Prony series dominating the relaxation response. The mechanical properties of brain tissueare listed in Table 2 [26].

**Table 2:** Material properties of the brain tissue [26].

Void ratio	Poisson’ ratio	Permeability M^4^/N s	Elastic Modulus (Pa)	$g_{k}^{p}$	*τ*_1_ (s)	*τ*_2_ (s)	*τ*_3_ (s)
0.2	0.35	4.08x10^-12^	350	0.285	3.1	27	410

The CSF is produced mainly in lateral, third and fourth ventricles [11,30]. As the greatest CSF production occurs in the lateral ventricles [30], the inlet flow location in this study, according to the study by Gholampour et al., was considered to be in the lateral ventricles [11]. The frequency and movement pattern of the inlet CSF flow rate function were obtained by normalizing the blood flow rate function in basilar artery and its amplitude was determined using the physiological data of CSF production [31]. Cine-PCMRI was used to measure the blood flow rate in the basilar artery and MATLAB R2013a software was applied for normalizing the blood flow rate diagram. Based on physiologic data, the total CSF production was set to 0.35 ml/min [30,32]. Accordingly the amplitude of the CSF inlet flow rate function was 0.35 ml/min. Finally, the inlet flow in this problem was obtained by superposing two diagrams in MATLAB: the diagram of the normalized blood flow rate (for frequency and the movement pattern of the inlet flow rate function) and the diagram of the constant amplitude (0.35 ml/min).

Two locations were considered for CSF outlets in this simulation: the sagittal sinus and spinal cord [2,30,32–36]. In reality, however, a little amount of CSF is drained through other parts in SAS and ventricular system that these outlets were neglected due to their low impact according to previous studies [2,11,35]. The amplitudes of the CSF outlets flow rate functions in sagittal sinus and spinal cord were 0.18 and 0.17 ml/min, respectively [36]. Considering that the amplitude of the inlet CSF flow rate function was assumed 0.35 ml/min, thus the net flow in each cardiac cycle was zero.

Similar to the inlet flow rate, the outlet flow rates of CSF were produced in MATLAB through superposing the diagrams of the normalized blood flow rate and the constant amplitude (0.18 ml/min for sagittal sinus and 0.17 ml/min for spinal cord).

The post-shunting results were obtained by adding one shunt with inner diameter of 1.3 mm in three-dimensional model of the patients’ right lateral ventricle and rerunning the models. The post-shunting boundary conditions were assumed similar to the pre-shunting conditions. Only the minimum and maximum values of the output pressure diagram in the shunt tube were considered 1422 and 1667 Pa, respectively. For more details about this pressure diagram see: Medtronic, Strata Various Adjustment System, Minnesota, USA.

**Fig 2:**
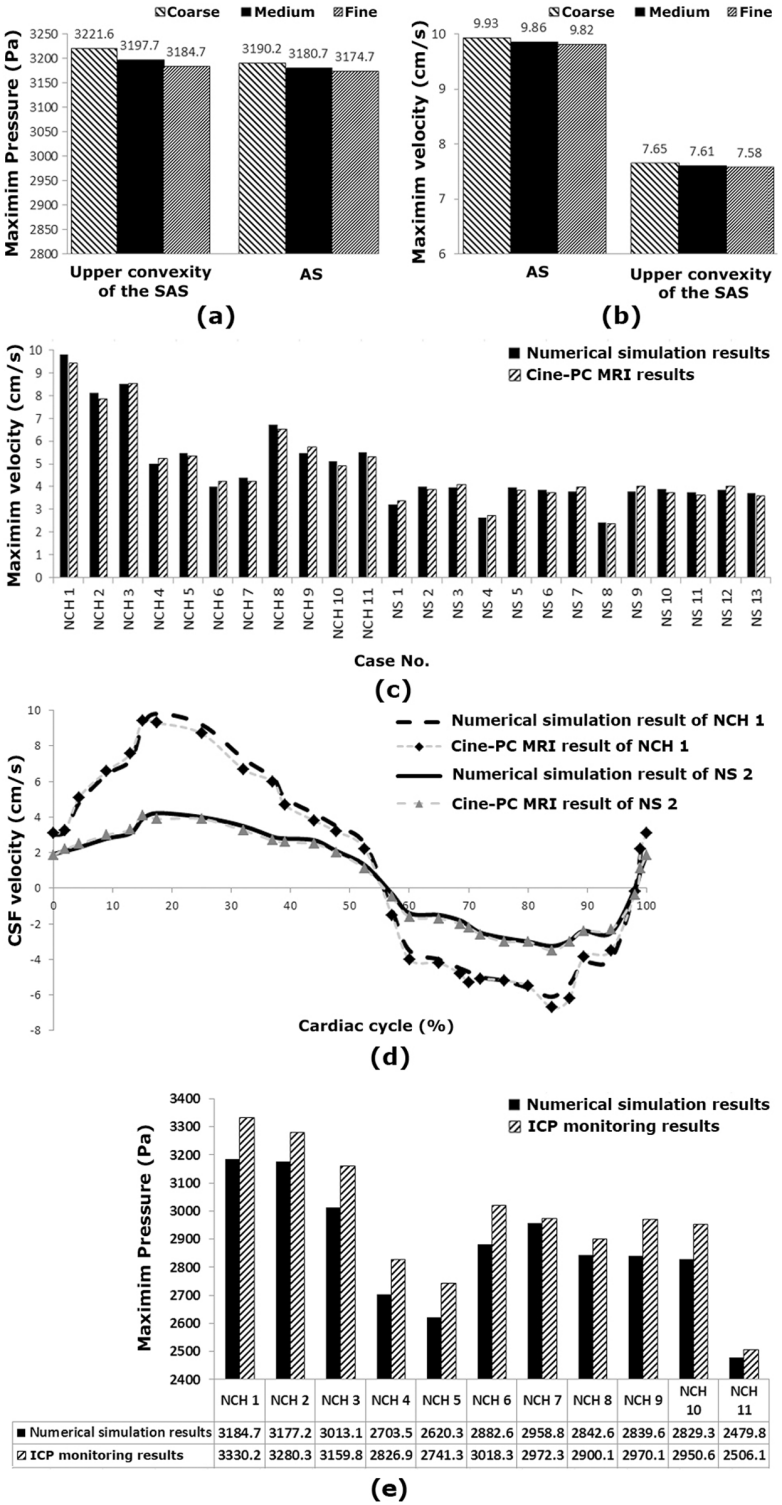
(a) and (b) show the three grid sizes used in the mesh independence study for maximum CSF pressure and maximum CSF velocity in aqueduct of Sylvius and upper convexity of the subarachnoid space for NCH patient No.1 before shunting, respectively. (c) shows the comparison of the maximum CSF velocities measured by cine-PC MRI in aqueduct of Sylvius and the maximum CSF velocities in aqueduct of Sylvius calculated by the FSI simulation for all 13 normal subjects and 11 patients. (d) shows the comparison of the cine-PC MRI velocity diagram in aqueduct of Sylvius and the velocity diagram calculated by the FSI simulation for the normal subject No. 2 and NCH patient No.1 before shunting. It is worth mentioning that the NCH patient No.1 before shunting and normal subject No. 2 have the highest velocity. (e) shows the comparison of the maximum ICP measured by ICP monitoring and the maximum ICP calculated by the FSI simulation for all 11 patients before shunting. SAS: subarachnoid space; AS: aqueduct of Sylvius; CSF: cerebrospinal fluid; NS: normal subject; NCH: non-communicating hydrocephalus; ICP: intracranial pressure.

Grid and time-step independence studies were crucial to ensure the accurate solutions of the applied numerical simulations. A fully implicit Euler scheme was implemented in this study. The initial grid was refined using the maximum simulated velocity and pressure at two locations by a step-size of 0.01s (Fig 2a and 2b). The maximum mesh intensity before and after shunting was created in AS. The numbers of original meshes of ventricular system and brain tissue in patient No. 1 were 1,217,308 and 762,104, respectively. Similar numbers of the same patient on day 5 after shunting were 1,020, 182 and 695, 819, respectively. Similar numbers for the same patient on day 18 after shunting were 1,007,214 and 687,105, respectively, and the same numbers on day 84 after shunting were 985,873 and 672,541, respectively, and the same numbers for the same patient on the day 903 after shunting were 981,203 and 667,357, respectively. The same numbers for the same patient were 981,019 and 667,317 on the day 981 after shunting. The entire process was repeated with a different initial grid for the coarse, medium and fine grids. The maximum percentage of difference between the medium and fine meshes for all 11 patients before and after shunting and 13 normal subjects was less than 0.39% (Fig 2a and 2b). No significant difference was observed in numerical simulations with smaller step sizes. Therefore, the grid independence study was valid for all cases.

### 2.4. Statistical analysis

Statistical parameters such as mean value, range, standard deviation (SD), coefficient of variation (CV) (standard deviation divided by the mean value) and Pearson correlation coefficient (PCC) were calculated using SPSS version 20.

## 3. Results

The results of 3D FSI simulation were calculated for each case and for each phase (one phase pre-shunting and five phases post-shunting) in 4 working cycles. Since there is no difference between the results of cycles 3 and 4, only the results of the third cycle are reported in this section. It is however necessary to guarantee the accuracy of boundary conditions, the assumptions and problem-solving process before examining the results through data validation.

### 3.1. Data validation

To ensure the validity of results, the CSF velocity in AS of all normal subjects and patients (before shunting) and the intracranial pressure (ICP) in patients (before shunting) were measured experimentally and compared with the computer simulation results. To do so, the velocity diagram of CSF in AS obtained from cine-PC MRI was compared with the one calculated by FSI modeling in all normal subjects and patients (Fig 2c and 2d). The reason for comparing the velocity of CSF in AS is related to the fact of having the least cross-section in the whole ventricular system and as a result, the highest velocity according to continuity law.

The maximum difference between the extremum (maximum or minimum) values of CSF velocity in AS measured by cine-PC MRI and CSF velocity calculated using FSI simulation for all cases was less than 3.8% while the maximum error in phase lag of these diagrams was less than 0.6% (Fig 2c and 2d).

To validate the CSF pressure results, the maximum ICP was measured in 11 patients (before shunting) using ICP monitoring. For this aim, a small opening was created in skull and in the dura and an ICP micro-sensor (Codman MicroSensor, Johnson and Johnson, Raynham, Massachusetts, USA) was inserted 1–2 cm into the brain parenchyma; the sensor was zeroed before insertion against the atmospheric pressure [37]. To obtain the ICP using FSI method, the maximum CSF pressure in the upper convexity of the brain in SAS was calculated at the output of the software.

As seen in Fig 2e, the maximum difference between the maximum ICP values measured by ICP monitoring and the maximum ICP calculate through FSI modeling in similar cases was less than 4.8%. The validation of CSF velocity and pressure results showed that there is a relative favorable agreement between the simulation results and experimental data. Further, it should be noted that all data presented in the Result sections 3.2–3.5 for patients are related to their conditions before shunting and all data included in the Result sections 3.2–3.5 and Discussion section have been calculated by computer simulation.

### 3.2. Geometrical properties of models

In order to evaluate the conditions of hydrocephalus patients, the changes in the volume of head substructures should be emphasized [38]. The mean volume of lateral, third and fourth ventricles in patients were 22.4, 2.5 and 1.2 times the similar volumes in normal subjects, respectively (Table 1). The acute increase in patients’ ventricular system volume (13.8 times) was related to the occurrence of hydrocephalus. Based on the results, the maximum volume changes occurred in lateral ventricles. We should expect a decrease in brain volume regarding the acute increase in CSF space volume. The mean brain volume of patients was 11.2% smaller than that of normal subjects (Table 1). Further, the mean volume of the SAS in patients was 3.1% higher than that of normal subjects. The volumetric change in the SAS was less than that in other parts during NCH disease progression.

### 3.3. Predicted CSF flow

**Fig 3:**
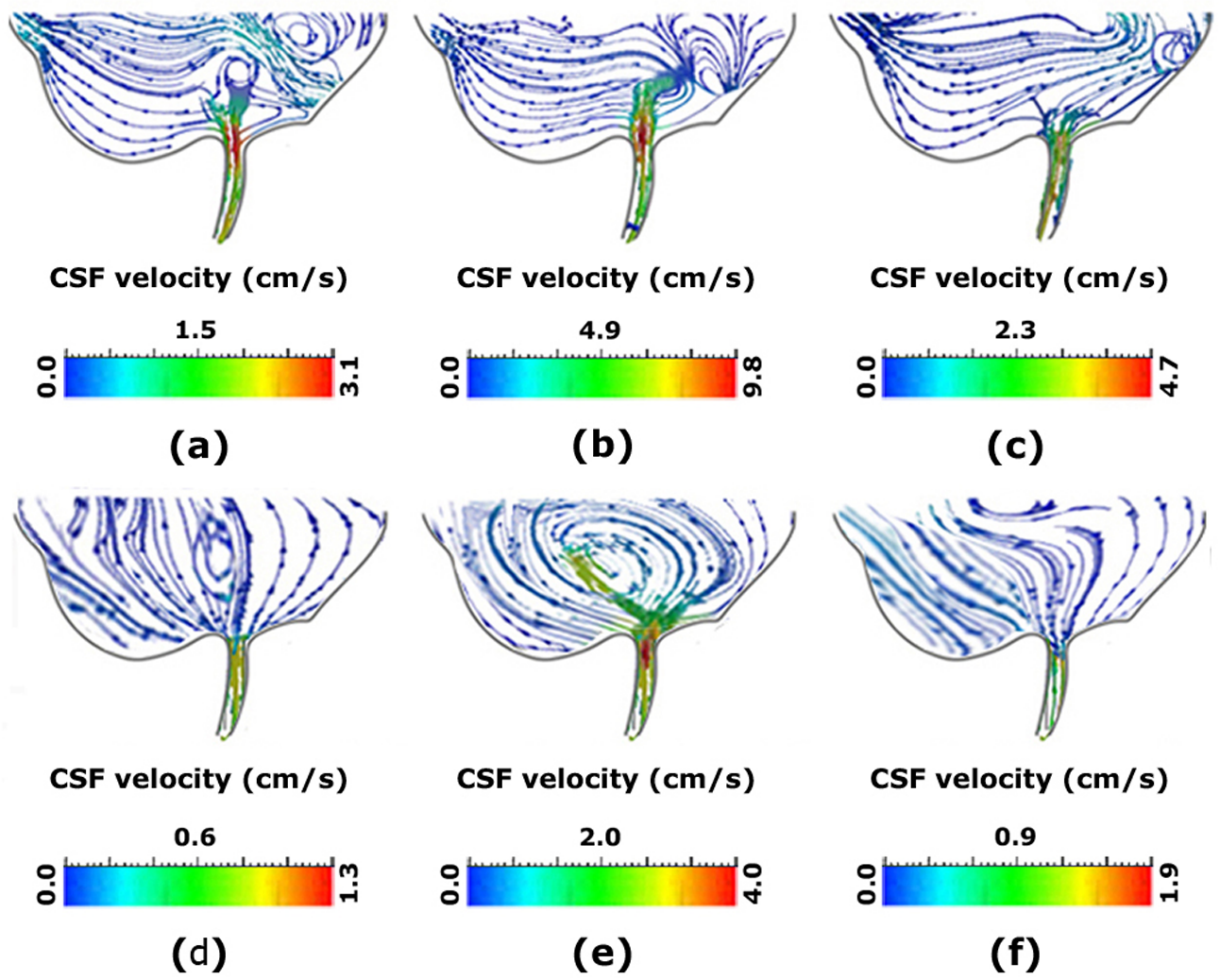
(a)—(c) and (d)–(f) show the vortex in the inferior section of third ventricle of NCH patient No.1 before shunting and normal subject No. 2, respectively. (b) and (e) show the vortex during the fill period. (c) and (f) show it during the flush period. It is worth mentioning that the NCH patient No.1 before shunting and normal subject No. 2 have the highest CSF velocity. The size of all panels is free not-to-scale. CSF: cerebrospinal fluid.

The precise examination of the details related to CSF velocity fields indicated irregular flow patterns in CSF flow. The results showed that the vortices were significantly weaker in normal subjects (Fig 3). Therefore, NCH could intensify the vortex. The pre-shunting location of vortex formation in NCH patient No. 1 was displaced toward the right side during the transition from Fig 3a to 3b (fill period) and to Fig 3c (flush period). However, no significant changes were observed in the location of vortex formation in normal subject No. 1 (Fig 3d–3f). Accordingly, the location of vortex formation and intensity of vortex involved a significant difference among the NCH patients and normal subjects.

**Fig 4:**
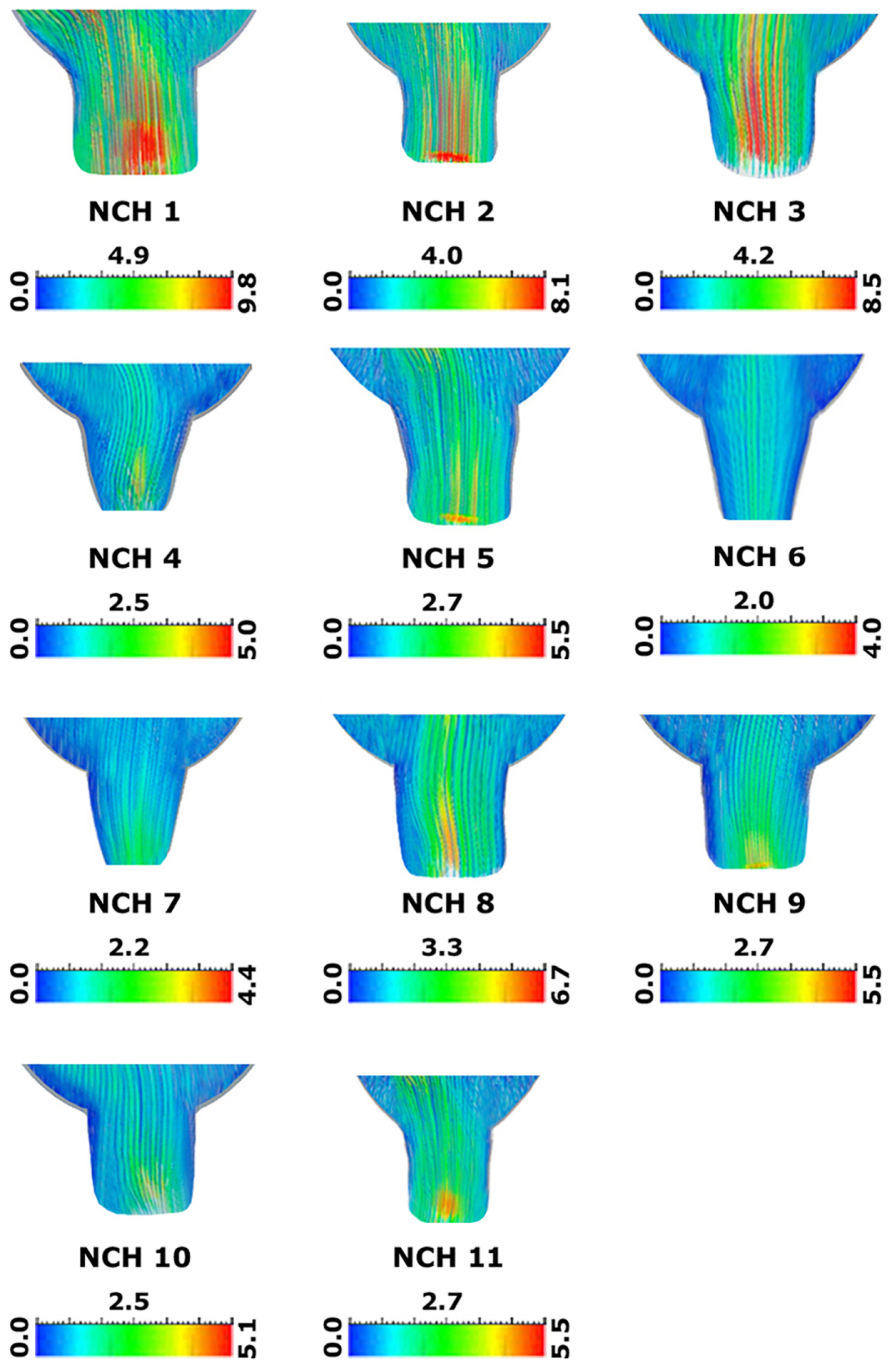
Compares the distribution of CSF velocity (cm/s) in the aqueduct of Sylvius that has the maximum velocity, among all 11 patients at the mid-systole. NCH: non-communicating hydrocephalus.

Based on the results in Fig 2d, the maximum and minimum CSF velocities occurred at 17.5% (mid-systole) and 84% (early systole) of the cardiac cycle, respectively. Fig 4 compares the CSF velocity distributions in the AS that has the maximum velocity, among all 11 patients at mid-systole. Cerebral vasculature expanded during systole and a compression of lateral ventricles was observed and CSF flowed into ventricular system and SAS during the early systole. Finally, CSF flowed into the spinal canal during the mid-systole and the later stages of systole.

The mean value of maximum CSF velocity value in AS was 6.20 ± 1.9 cm/s among the 11 patients while it was 3.92 ± 1.3 cm/s for the 13 normal subjects (Table 1). The mean value of maximum CSF velocity value in AS among the patients was 58% more than that of the normal subjects. The Reynolds number is the ratio of inertial forces to viscous forces [39] and is considered as the best index to determine whether the CSF is laminar or turbulent. Based on the diameters and CSF velocities of aqueduct, the mean value of maximum Reynolds number among patients was 22% more than that of the normal subjects (Table 1). Accordingly, the CSF flow was still laminar after NCH occurrence despite of the slight increase in Reynolds number.

### 3.4. Predicted CSF pressures

Generally, the clinical symptoms of NCH are divided into two groups [11]. First, the clinical symptoms like loss of coordination or balance, which is mainly related to increase in CSF pressure in ventricles. Hence, the changes of these symptoms are represented by CSF pressure in AS [11]. Second, the clinical symptoms like papilledema and impaired vision which are mainly related to an increase in CSF pressure in BONS [11]. Therefore, evaluating CSF pressure, especially in AS and BONS, is emphasized during analysis of NCH patients. On the other hand, obtaining information on the amount of CSF pressure in SAS and lateral ventricles can be very useful for calculating the transmittal pressure gradient.

The patients’ mean of maximum CSF pressure in the lateral ventricle, AS, BONS and the maximum ICP are 2853.6 ± 215.8, 2847.2 ± 215.6, 2856.5 ± 218.2 and 2866.5 ± 216.2 Pa, respectively (Table 3). Therefore, the mean value of maximum CSF pressure in NCH patients is about 5.1~5.3 times the corresponding pressures in normal subjects. The mean value of CSF pressure amplitude in the lateral ventricle, AS and BONS and the mean value of ICP amplitude in patients are 136.2 ± 10.1, 131.5 ± 9.0, 147.7 ± 10.7 and 141.3 ± 10.3 Pa, respectively (Table 3). The maximum CSF pressure amplitude in all cases dedicated to the BONS and the mean value of CSF pressure amplitude in patients is 2.3~2.4 times the normal subjects.

**Table 3:** CSF pressure (Pa) details of the 11 NCH patients pre-shunting and mean values of CSF pressure of the 13 normal subjects. The amount of pressure amplitude is maximum pressure—minimum pressure. The Womersley numbers have been also plotted in the inferior section of third ventricle. NCH: Non-communicating hydrocephalus; NCH: Non-communicating hydrocephalus; SAS: subarachnoid space; BONS: behind optic nerve sheath; AS: aqueduct of Sylvius; LV: lateral ventricle.

Case No	NCH 1	NCH 2	NCH 3	NCH 4	NCH 5	NCH 6	NCH 7	NCH 8	NCH 9	NCH 10	NCH 11	13 NS Mean±SD
MaximumCSF pressure -SAS	3184.7	3177.2	3013.1	2703.5	2620.3	2882.6	2958.8	2842.6	2839.6	2829.3	2479.8	560.9.±31
Amplitude of the CSF pressure -SAS	155.8	142.5	140.8	142.8	149.1	140.6	114.0	140.6	145.7	144.5	138.7	59.6.±4.2
MaximumCSF pressure -BONS	3181.9	3174.8	3006.7	2691.7	2614.1	2868.3	2945.1	2828.3	2823.9	2816.3	2470.6	557.4±31
Amplitude of the CSF pressure -BONS	155.8	145.9	156	147.4	157.3	140.5	121.6	140.5	154.2	156.8	148.9	64.5±4.4
MaximumCSF pressure -AS	3174.7	3156.8	3001.4	2687.3	2603.7	2864.9	2922.3	2824.9	2800.7	2813.4	2468.7	540.8.±32
Amplitude of the CSF pressure -AS	122.6	136.8	130.4	128.9	131.6	115.1	150.2	132.4	139.2	132.7	126.9	54.0±3.9
MaximumCSF pressure -LV	3174.5	3155.9	3000.2	2686.4	2603.3	2863.8	2941.6	2823.8	2859.1	2812.6	2467.8	535.0±32
Amplitude of the CSF pressure -LV	138.2	156.5	138.2	147.1	126.7	134.5	142.6	134.5	128.4	132.1	119.6	57.3±4.0
Womersleynumber	6.8	7.3	7.1	6.9	7.1	6.2	8.9	7	7.6	7.1	6.9	3.0±0.2

### 3.5. Pulsatility of CSF flow

The Womersley number was used to analyze the pulsatility of CSF flow. The Womersley number is regarded as a non-dimensional number derived from the unsteady Navier-Stokes equations and an expression of the pulsatile flow frequency in relation to viscous effects and is calculated by the following equation [40]:

$$\alpha =R\sqrt{\frac{2\pi \rho^{f}}{T\mu }}$$ (6)

where α represents the Womersley number, T indicates the time period of the cardiac cycle and R shows the radius of aqueduct.

The Womersley number was greater than 1 for all cases (Table 3). The flow pattern was not parabolic as α>1 [40]. The mean value of Womersley number in the inferior section of the third ventricle was 7.2 ± 0.7 and 3.0 ± 0.2, respectively, for patients and normal subjects. The results indicated an increase in CSF flow pulsatility by 2.4 times after occurrence of NCH.

**Fig 5:**
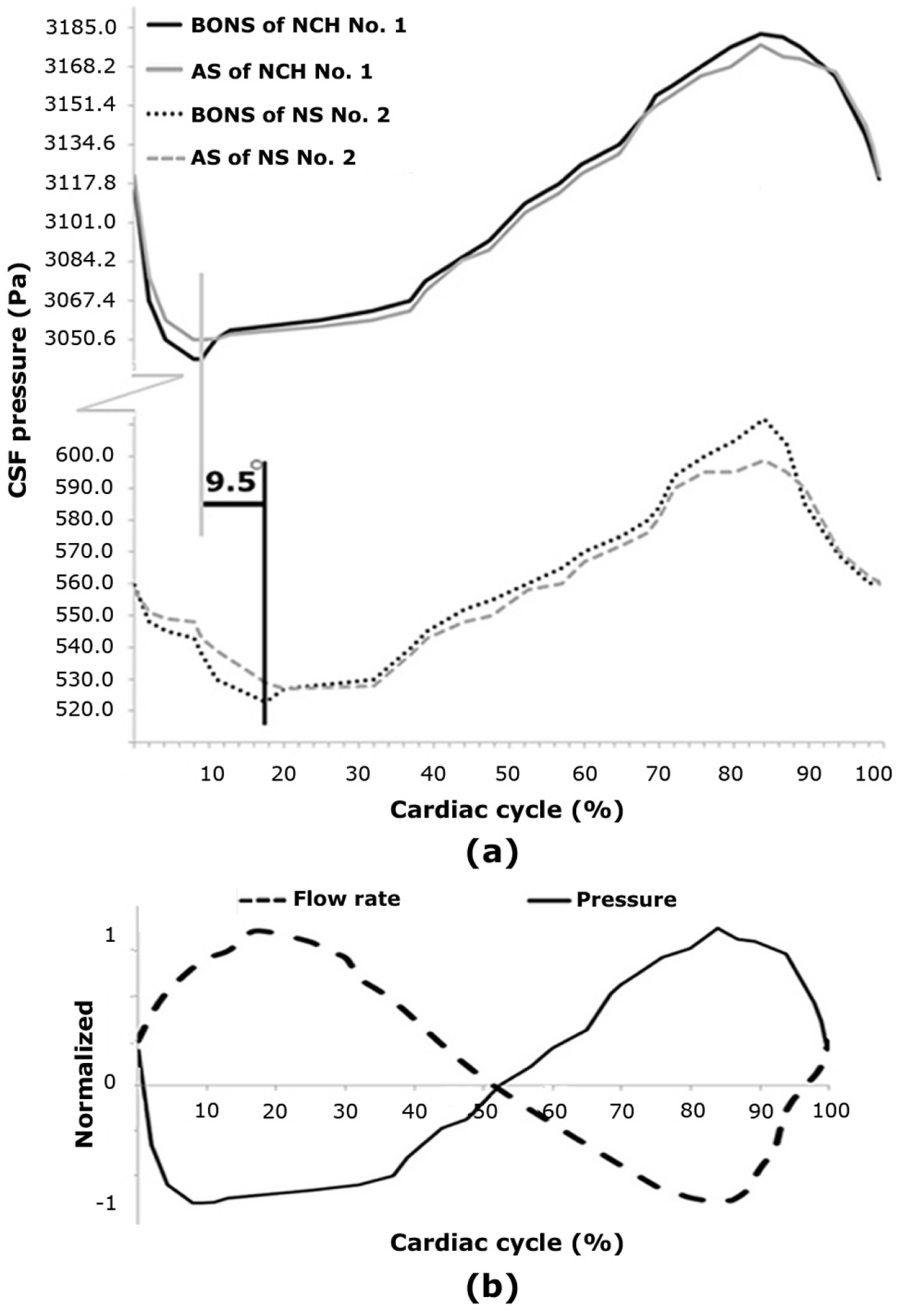
(a) Compares pressure diagrams in the aqueduct of Sylvius and behind the optic nerve sheath of NCH patient No.1 before shunting and normal subject No.2. There is 9.5° phase lag between occurrence time of minimum pressure in the patient and the normal subject. It is worth mentioning that the NCH patient No.1 before shunting and normal subject No. 2 have the highest CSF pressures. (b) Shows normalized flow rate and pressure gradient diagrams in the aqueduct of Sylvius of patient No.1. The phase lag of flow rate and pressure gradient diagrams in the aqueduct of Sylvius of the NCH patient No.1 before shunting is 59.5°. AS: aqueduct of Sylvius; BONS: behind optic nerve sheath; CSF: cerebrospinal fluid; NCH: non-communicating hydrocephalus; NS: normal subject.

The CSF pressure diagrams of the NCH patient No. 1 before shunting and the normal subject No. 2 were compared with each other in two cross-sections of the AS and BONS (Fig 5a). Contrary to velocity diagrams, the minimum pressure occurred at 17.5% of the cardiac cycle in normal subjects while it happened at 8% of the cardiac cycle among the patients (Fig 5a). Furthermore, the maximum pressure occurred at 84% of the cardiac cycle in both patients and normal subjects.

Womersley proved a phase lag between flow rate and pressure gradient functions [41]. Fig 5b illustrates this phase lag in the AS of the NCH patient No. 1 (before shunting) during a cardiac cycle. The mean value of this phase lags in patients and normal subjects were 57.6° ± 5.1° and 48.2° ± 3.2°, respectively.

## 4. Discussion

In the present study, the differences between CSF bio-fluid parameters in patients (before shunting) and normal subjects were compared first to find the proper hydrodynamic indices before shunting. Then, the conditions of three patients were followed up until 981 days after shunting, but evaluation of the changes in cellular response of NCH brain tissue was not the objective of this study.

### 4.1. Proper index for evaluation of NCH patients before treatment

In this section, the conditions of patients (before shunting) and normal subjects are compared. First, it seems necessary to investigate and evaluate the data dispersion for each parameter statistically.

The CV for the maximum CSF velocity of NCH patients was more than 30% while it was less than 7.6% and 6.5% for the maximum CSF pressure and ventricles volume of the same patients, respectively (Tables 1 and 3). Therefore, the velocity data were more dispersive than other data in the same conditions. The results of the previous studies indicated a very wide range of values (1.67~16.7 cm/s) for maximum CSF velocity in the AS of normal subjects [1,10,11,13,42,43]. The velocity results of the present study confirmed also a similar variety among both normal subjects and patients (Table 1 and S1 Appendix). Moreover, the value of maximum CSF velocity in the AS was almost equal in normal subject No.2 and NCH patient No. 6 (before shunting) (Table 1 and S1 Appendix). In addition, the results indicated that the mean value of maximum CSF velocity in patients was only 1.58 times the normal subjects. However, the mean value of maximum CSF pressure and ventricles volume in NCH patients was more than 5.1 and 13.8 times that of the normal subjects, respectively (Tables 1 and 3). Therefore, these comparisons before shunting show that the values of maximum CSF pressure and ventricles volume are more consistent and accurate, and these parameters are accordingly more proper indices, than the velocity, for hydrodynamic assessment of NCH patients. The clinical symptoms of patients before shunting are listed in Table 1. Although the conditions of the data dispersion and mean value of the Womersley number are also acceptable, the Womersley number is not an independent parameter for NCH hydrodynamic assessment and its concept depends on other parameters.

**Fig 6:**
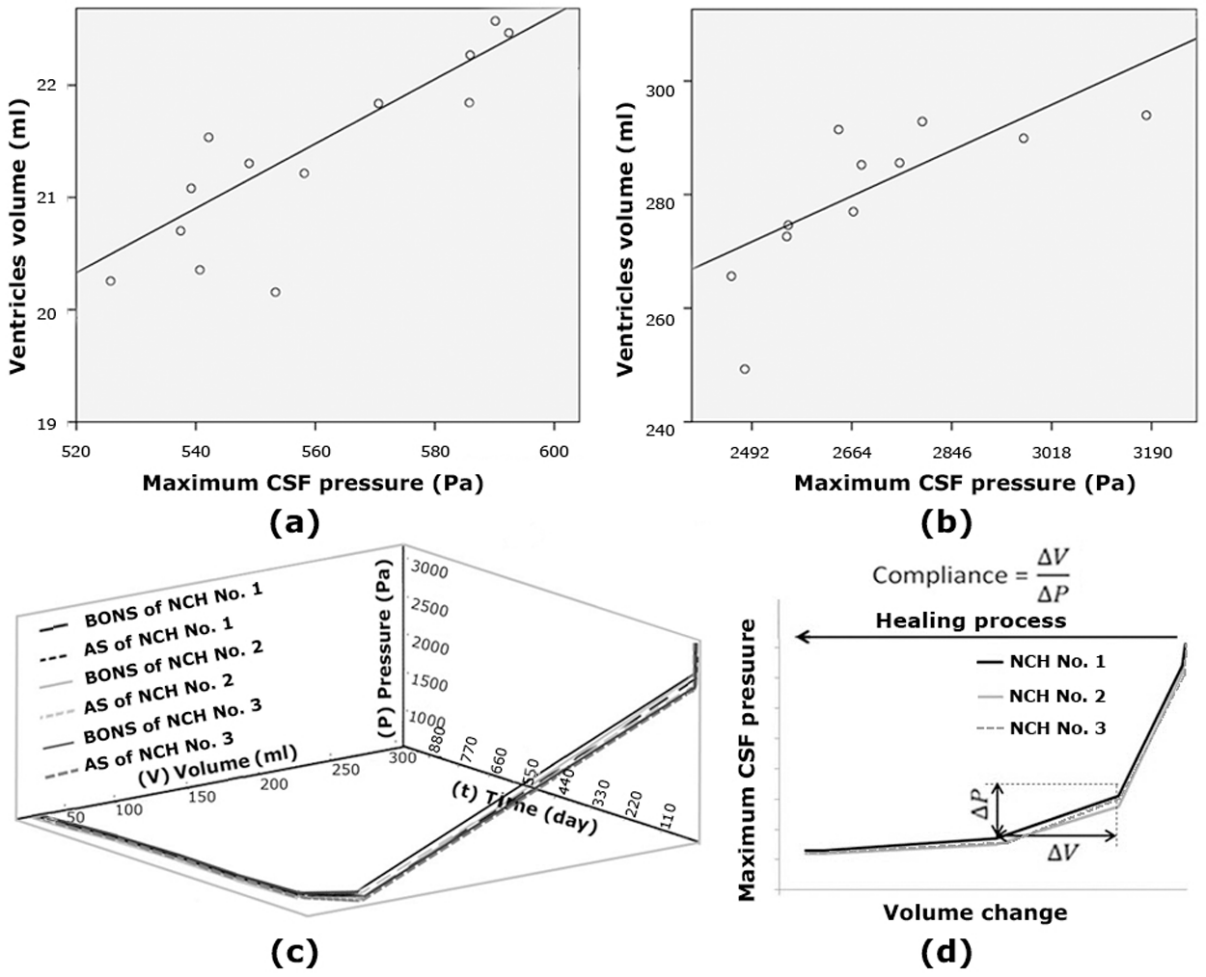
(a) and (b) show correlation between maximum CSF pressure and the ventricles volume in 13 normal subjects and 11 patients (before shunting), respectively. (c) shows maximum pressure-ventricles volume-time (P-V-t) diagram in the aqueduct of Sylvius and behind optic nerve sheath of patients No.1, 2 and 3 after shunting. This diagram has three views: maximum pressure-time (P-t), ventricles volume-time (V-t) and maximum pressure-ventricles volume (P-V). P-t and V-t views of this diagram clearly show the manner of reduction of volume and pressure during time in patients No. 1–3. (d) shows intracranial compliance of patients No. 1–3 during healing process. The units of pressure and volume are Pascal and millilitre, respectively. AS: aqueduct of Sylvius; BONS: behind optic nerve sheath; NCH: non-communicating hydrocephalus; CSF: cerebrospinal fluid.

The PCC represents a measure for the relationship between two variables. The range of this coefficient is +1 to -1. The PCC between proper indices (maximum CSF pressure and the ventricles volume) was +0.91 (p<0.01) and +0.84 (p<0.01) for normal subjects and patients, respectively (Fig 6a and 6b). PCC results confirmed an appropriate correlation between two proper indices although the correlation decreased slightly after the occurrence of disease. In other words, the changing trend of these two indices was not similar in patients and normal subjects.

Based on the results, the difference between proper indices in the three groups of patients (Table 1) including aqueductal stenosis, mesencephalic tumour and aqueductal web is less than 7.6% (Tables 1 and 3). Thus, the cause of NCH does not considerably affect the manner of changes in these proper indices. In future studies, Tables 1 and 3 should be extended by both greater number of patients and more diverse groups of NCH patients regarding age, gender and causes of NCH in order to evaluate this effect more accurately.

### 4.2. CSF hydrodynamic changes and the NCH effects in treatment processes

All the aforementioned steps from MRI to meshing and analysis were repeated for new head models of NCH patients No.1-3 at five post-treatment stages within 5, 18, 81, 903 and 981 days after shunting. Unfortunately, follow-up of other patients was not possible.

The maximum CSF pressure in both BONS and AS of NCH patient No.1 within 5, 18, 81, 903 and 981 days after shunting was about 48.0%, 81.0%, 81.9%, 81.9% and 81.9% less than the pre-shunting pressures, respectively (Fig 6c and Table 3). Further, the ventricles volume of NCH patients on these days was 54.4%, 89.6%, 91.2%, 91.5% and 91.5% less than the pre-shunting volumes, respectively (Fig 6c and Table 1). In addition, the reduction of values of volume and maximum pressure in NCH patients No. 2 and 3 was also approximately similar to that of NCH patient No.1 with a difference of less than 1.9% (Fig 6c).

Based on report of the attending medical team, all clinical symptoms of the disease except for headache disappeared completely in all three patients within 18 days after shunting. The maximum CSF pressure in both BONS and AS returned to mean values of these pressures in normal subjects with a difference of merely 8.7~8.9%. For instance, NCH patients No. 1–3 and 7 experienced papilledema symptom before shunting (Table 1) but this disappeared in NCH patients No. 1–3 on the day 18 simultaneous with a significant decrease in their BONS pressures. It should be emphasized that headache symptoms didn’t disappear on day 18 although their severity was significantly dropped compared to day 5. The ventricles volume of patients on day 5, despite of its 54.4% reduction, was still about 6.4 times of the mean value of ventricles volume among normal subjects. This extra volume is still considerable in comparison to normal subjects. On day 18, the ventricles volume of patients experienced more reduction (89.6%) and this was less than 1.5 times of the mean value of ventricles volume among normal subjects. The headache of patients was relieved significantly on day 18, however, the headaches were not eliminated completely. However, the pressure values returned in a satisfying manner to the pressure range of normal subjects within 18 days; they were merely less than 8.9% greater than the mean value of pressure in normal subjects. Results also showed that the amounts of Womersley number in NCH patients No. 1–3 were 6.5, 6.9 and 6.8, respectively. These values indicate a change of less than 4.8% in Womersley numbers.

On day 81, the maximum pressure values in BONS and AS of NCH patient No.1 reduced to 590.6 Pa and 572.4 Pa, respectively. The ventricles volume was about 23.3% greater than the mean value of ventricles volume in normal subjects. The results of models analyses on day 81 showed that the values of Womersley number were almost constant with a difference of less than 1.6% comparing to day 18. Although pressure values reached very close to normal conditions and all the clinical symptoms vanished on day 81, the ventricles volumes and the flow pulsatility (Womersley number) had still a significant difference comparing to normal subjects’ conditions.

On day 903, the ventricles volume among the patients decreased by about 3.2% (about 20.1% more than the mean value ventricles volume in normal subjects) although the maximum pressures in BONS and AS were equal to the pressure values calculated on day 81. In other words, the CSF pressure reached a stable and constant state on day 81 after shunting while the ventricles volume reduction in patients continued on a slight slope. The value of Womersley number had no significant changes between days 81 and 903 after shunting. Further, no changes were observed in all examined parameters between days 903 and 981 after shunting. It is worth mentioning that the phase lags between the flow rate and pressure gradient functions had no changes from pre-shunting step up to 981 days after shunting.

The important point is that even more then 2.5 years after shunting and complete healing of patients the numerical values of none of the examined parameters, except for pressure, returned to the ranges in normal subjects. For example, the results obtained for Womersley number showed that the average of this number after occurrence of NCH increased 2.4 times but the level of CSF flow pulsatility had no significant decrease (less than 6%) after complete healing of patients.

Furthermore, NCH caused permanent volume changes in ventricular system and this volume change continued even 2.5 years after shunting. It can therefore be concluded that about 20.1% of the ventricles volume likely wouldn’t return to a normal state more than 2.5 years after shunting and healing. In other words, the amount of 20.1% can be equivalent to the concept of residual strain since this amount is related to the volume, which has not returned to mean value of ventricles volume in normal subjects even more than 2.5 years after unloading of brain tissue and ventricles through shunting.

Despite the fact that clinical symptoms in patients disappeared on day 81 after shunting, NCH caused permanent changes in parameters such as ventricular volume, Womersley number and phase lag between flow rate and pressure gradients and shunt surgery and treatment process could not play any role in changes of these parameters. It can be deduced that despite of obvious difference in many of CSF bio-fluid parameters with respect to normal subjects, after shunting, patients have experienced a new healthy state in new hydrodynamic conditions different from normal conditions.

Moreover, P-t and V-t views in Fig 6c can help in prediction of patients’ recovery time, which would be more accurate by further development of this simulation through recruitment of patients with a greater variety of age and sex. Reduction of maximum pressure and volume change during the healing process of NCH patients is associated with an increase in intracranial compliance (ICC) (Fig 6d). ICC is defined as the ratio of maximum pressure and volumechange, ΔV/ΔP [44] and is an effective parameter in diagnosis and treatment of hydrocephalus patients [11,44]. Most of the previous methods used to find the ICC diagram were mainly invasive while a non-invasive method was implemented in the present study. Further, the viscous character and time-dependent property of brain tissue was considered in a more effective manner in the ICC diagram calculations of the present study. In previous studies, the volume and pressure changes and ICC diagram were measured within a short period of time after shunting while in this study, they were calculated up to more than 2.5 years after shunting and after complete stabilization of volume and pressure changes. Hence the trend of the ICC diagrams of previous experimental studies differ from that in the present study [44,45].

### 4.3. Model limitations and future work

In the present study, the white and gray matters were not considered separately in the brain tissue model. Of course, the brain tissue has been considered as a single part in many studies and there are also conflicts about the importance and necessity of modeling the brain in two parts. On the one hand, according to the study by Dutta-Roy et al., a single-part-model is also sufficient for modeling the brain in patients with normal pressure hydrocephalus [46]. On the other hand, according to the study by Tavner et al., using a two-part-model is further justify for the brain tissue analysis in general [47]. It is hence suggested to consider these two parts separately in brain modeling in future studies. It is also suggested to perform a similar research in the future studies on patients treated by ETV method and to compare the results of VPS and ETV methods in order to assess the effectiveness of the two treating methods.

## 5. Conclusion

The changes in CSF biofluid parameters during shunt treatment process of NCH patients using computer simulation hasn’t been investigated in previous studies. In the present study, these effects and ICC diagram have been investigated using 3D FSI simulation up to 2.5 years after shunt surgery in a large number of NCH patients through a non-invasive method. Results indicated that the ventricles volume and maximum CSF pressure are more proper indices, than the velocity, for hydrodynamic assessment of NCH patients (before shunting). The type of hydrocephalus also played no significant role in changing these indices. Moreover, the intensity of vortex increased, the location of vortex formation changed and the CSF flow was 2.4 times more pulsatile after NCH occurrence while the CSF flow remained laminar. The results also indicated that all clinical symptoms, except headaches, disappeared on 18 day after shunting. The ventricles volume of patients on day 18, despite of the considerable pressure drop (about 8.7~8.9% greater than the mean value of pressure in normal subjects), was 1.5 times more than the mean value of ventricles volume in normal subjects. Although investigating the biofluid parameters before the beginning of treatment process showed that the maximum CSF pressure and ventricles volume are the most proper bio-fluid indices, continuing this investigation during the treatment process showed that maximum CSF pressure is the most sensitive and the most proper hydrodynamic parameter. Maximum CSF pressure has decreased significantly, proportional for the level of decrease in clinical symptoms, and it has returned to a level close to normal subjects’ conditions. It also returns close to the pressure range in normal subjects faster than other parameters and simultaneous with disappearance of patients’ clinical symptoms (from day 81 after shunting) and its value remains stable and constant state on day 81 after shunting. However, NCH has caused permanent changes in phase lag between flow rate and pressure gradient functions, Womersley number (the level of pulsatility of CSF flow) and ventricles volume (about 20.1% more than the mean value ventricles volume in normal subjects) even 981 days after shunting and healing. Therefore, it can be deduced that patients have experienced a new healthy state in new hydrodynamic conditions after shunting and healing despite the fact that there are permanent changes in CSF biofluid parameters (except for pressure) and these parameters haven’t returned to normal subjects’ conditions. Finally, the manner ICC increases during the healing process of NCH has been presented in this study in a more precise way comparing to previous studies. The manner of changes in CSF biofluid parameters with regard to their clinical symptoms can reflect the complexity of CSF flow dynamics in NCH patients during healing process and can help physicians in quantitative assessment of NCH patients’ conditions and can also help to gain more insight into the pathophysiology of NCH patients.

## Supporting information

S1 Appendix Relevant information on 13 normal subjects: Gender, maximum CSF velocity (cm/s), Reynolds number, details of CSF pressure (Pa), head substructure’s volume (ml), and Womersley numbers. The Womersley numbers have been also plotted in the inferior section of third ventricle. SAS: subarachnoid space; BONS: behind optic nerve sheath; AS: aqueduct of Sylvius; LV: lateral ventricle; NS: normal subject.
(DOCX)
