# Supplementary material for: FSI simulation of CSF hydrodynamic changes in a large population of non-communicating hydrocephalus patients during treatment process with regard to their clinical symptoms
Source: PLoS One. 2018 Apr 30;13(4):e0196216. doi: 10.1371/journal.pone.0196216 (PMC5927404; doi:10.1371/journal.pone.0196216)
Supplement: S1 Appendix — The Womersley numbers have been also plotted in the inferior section of third ventricle. SAS: subarachnoid space; BONS: behind optic nerve sheath; AS: aqueduct of Sylvius; LV: lateral ventricle; NS: normal subject. (DOCX) [file pone.0196216.s001.docx]

S1 Appendix: Relevant information on 13 normal subjects: gender, maximum CSF velocity (cm/s), Reynolds number, details of CSF pressure (Pa), head substructure's volume (ml), and Womersley numbers. The Womersley numbers have been also plotted in the inferior section of third ventricle.

SAS: subarachnoid space; BONS: behind optic nerve sheath; AS: aqueduct of Sylvius; LV: lateral ventricle; NS: normal subject.

| Case number | NS 1 | NS 2 | NS 3 | NS 4 | NS 5 | NS 6 | NS 7 | NS 8 | NS 9 | NS 10 | NS 11 | NS 12 | NS 13 |
| --- | --- | --- | --- | --- | --- | --- | --- | --- | --- | --- | --- | --- | --- |
| Gender | Female | Male | Female | Male | Male | Female | Male | Female | Male | Male | Female | Female | Male |
| Volume - LVs | 11.8 | 12.5 | 11.7 | 10.9 | 11.64 | 10.9 | 12.4 | 11.6 | 11.7 | 12.3 | 11.9 | 12.4 | 11.4 |
| Volume -3V | 4.36 | 4.65 | 4.87 | 4.81 | 3.92 | 4.61 | 4.87 | 4.75 | 4.71 | 4.65 | 4.92 | 4.81 | 4.76 |
| Volume -4V | 3.96 | 3.85 | 4.1 | 3.65 | 4.03 | 3.57 | 3.96 | 3.65 | 4.09 | 3.94 | 4.11 | 3.96 | 3.81 |
| Volume -SAS | 108.5 | 104.2 | 107.3 | 103.7 | 106.8 | 104.1 | 109.2 | 115.6 | 102.3 | 103.1 | 100.6 | 102.9 | 98.6 |
| Volume -Brain | 1243.5 | 1206.8 | 1215.3 | 1242.3 | 1235.6 | 1208.9 | 1121.8 | 1295.3 | 1209.6 | 1198.3 | 1200.8 | 1095.5 | 1218.5 |
| MaximumCSF velocity -AS | 3.484 | 4.02 | 3.98 | 2.95 | 3.97 | 3.85 | 3.86 | 2.678 | 3.88 | 3.94 | 3.85 | 3.88 | 3.79 |
| Reynoldsnumber -AS | 335.2 | 350.8 | 318.2 | 260.5 | 335.2 | 274.6 | 337.2 | 276.4 | 295.3 | 259.4 | 340.6 | 342.6 | 314.2 |
| MaximumCSF pressure -SAS | 622.2 | 621.3 | 529.4 | 571.8 | 548.9 | 562.6 | 549.4 | 554.1 | 569.8 | 556.6 | 550.6 | 546.8 | 508.4 |
| Amplitude of the CSF pressure -SAS | 65.1 | 64.8 | 57.32 | 63.5 | 52.1 | 61 | 59.7 | 55.4 | 61.5 | 63.8 | 58.1 | 58.6 | 53.2 |
| MaximumCSF pressure -BONS | 621.5 | 612.1 | 528.3 | 568.3 | 547.6 | 559.2 | 548.9 | 550.2 | 564.8 | 546.3 | 549.9 | 541.6 | 507.6 |
| Amplitude of the CSF pressure -BONS | 62.5 | 79.7 | 63.8 | 64.3 | 63.6 | 64.4 | 63.9 | 68.1 | 64.7 | 61.6 | 65.1 | 59.7 | 63.8 |
| MaximumCSF pressure -AS | 611.3 | 581.4 | 518.2 | 553.6 | 538.7 | 541.2 | 534.2 | 512.8 | 541.6 | 540.6 | 541.1 | 539.5 | 475.9 |
| Amplitude of the CSF pressure -AS | 56 | 59.8 | 50.8 | 57.8 | 53.8 | 57.8 | 53.9 | 51.8 | 53.4 | 48.9 | 58.9 | 50.8 | 47.9 |
| MaximumCSF pressure -LV | 608.5 | 548.9 | 510.9 | 551.8 | 530.6 | 538.9 | 532.6 | 508.9 | 539.8 | 532.6 | 540.1 | 537.4 | 474.5 |
| Amplitude of the CSF pressure -LV | 58.5 | 61.7 | 54.1 | 59.8 | 65.3 | 60.1 | 58.6 | 56.7 | 49.5 | 55.9 | 56.3 | 54.8 | 54.3 |
| Womersleynumber | 3.1 | 2.8 | 3.2 | 3.1 | 3.2 | 2.7 | 2.9 | 3.4 | 3.2 | 2.7 | 2.9 | 2.7 | 2.8 |
